# Stereo‐Electroencephalography Reveals Dynamic Spatio‐Spectral Signatures of Negative Emotions: A Distributed Circuit Mechanism Involving Amygdala–Cingulate–Hippocampus–Orbitofrontal Networks

**DOI:** 10.1002/brb3.70951

**Published:** 2025-09-30

**Authors:** Zheng Li, Rong Yang, Jiaxi Zhao, Kanglin Liu, Sixun Yu, Xin Chen, Haifeng Shu

**Affiliations:** ^1^ Department of Neurosurgery Western Theater General Hospital Chengdu Sichuan Province China; ^2^ Department of Neurosurgery The Affiliated Hospital of Southwest Medical University Luzhou Sichuan Province China; ^3^ Tissue Stress Injury and Functional Repair Key Laboratory of Sichuan Province China

**Keywords:** functional brain network, negative emotion, stereo‐electroencephalography, time‐frequency analysis

## Abstract

**Background:**

The brain functional network connection mechanism mediating negative emotions is a central research focus in affective neuroscience. Although prior studies have identified key regions involved in emotion processing, the dynamic interactions among these regions remain inadequately understood.

**Methods:**

In this study, stereo‐electroencephalography (SEEG) was employed to simultaneously record local field potentials from both human cortical and subcortical structures. This technique enables the tracking of dynamic time‐frequency responses and functional connectivity patterns evoked by emotional stimuli with high spatial and temporal resolution.

**Results:**

The findings indicate that the amygdala (Amy), anterior cingulate cortex (ACC), hippocampus (Hip), and orbitofrontal cortex (OFC) play pivotal roles in negative emotion processing. Importantly, the experience of negative emotions depends on dynamic network interactions among these regions.

**Conclusion:**

This study provides direct electrophysiological evidence supporting previous investigations of emotional neural circuits based on electroencephalography (EEG) and functional magnetic resonance imaging (fMRI). Furthermore, it offers novel insights into the real‐time network mechanisms underlying emotion processing.

**Trial Registration:**

Chinese Clinical Trial Registry: ChiCTR2400080217

## Graphical abstract

This study innovatively integrated stereotactic electroencephalography (SEEG) with standardized visual emotion paradigms, systematically elucidating the dynamic time‐frequency characteristics and functional connectivity patterns within negative emotion‐related brain networks.

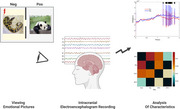

## 1 Introduction

Emotion, as an integrative state encompassing both physiological and psychological processes, constitutes a dynamic system of multidimensional elements. This system emerges from interaction between subjective experience and objective environment, intertwining with cognitive processes, physiological changes, and adaptive behavior (Xu et al. 2017). Beyond a direct reaction to external stimuli, emotion functions as a dynamic evaluative mechanism for both the individual and their environment. At its core, emotion represents a physiological marker linked to reward or punishment, primarily facilitating environmental adaptation through behavioral regulation. However, dysregulated anxiety or fear—whether excessive or insufficient—can impair threat responsiveness, as seen in anxiety disorders, depression, and bipolar disorder (Xin et al. 2020). Effective regulation of negative emotions (e.g., fear, anxiety) is thus critical for mental health, well‐being, and social functioning.

Functional differentiation and integration are two fundamental principles governing brain network organization. Anatomically distinct regions specialize in specific neural functions. Upon encountering a salient stimulus, the nervous system initiates cascades of cognitive processes, driving large‐scale recruitment and dynamic reorganization of functional brain networks (Fang et al. 2020). The amygdala (Amy), a key node in the limbic system, plays a central role in processing negative emotions, behavioral regulation, and emotional information. The Amy interacts extensively with sensory regions (e.g., the visual cortex) via neural pathways, enabling rapid stimulus‐response integration. During emotional processing, distributed brain regions—including the Amy, orbitofrontal cortex (OFC), medial prefrontal cortex (mPFC), anterior cingulate cortex (ACC), Insular lobe (Ins), hippocampus (Hip), and others—collaborate to form a dynamic network (Silverman et al. 2019). This network supports emotion encoding, memory consolidation, and decision‐making. Distinct emotional states (e.g., fear, anger) activate unique combinations of regions, yielding specific neural patterns. Investigating these patterns provides insights into functional specialization and interregional interactions.

Despite advances in electroencephalography (EEG) and functional magnetic resonance imaging (fMRI), limitations persist in capturing the transient dynamics of emotional processing. fMRI's temporal resolution is constrained by hemodynamic delays (∼2–6 s), while EEG suffers from poor spatial accuracy (∼5–10 mm errors) due to volume conduction (W. Li et al. 2022). Although EEG‐fMRI fusion studies have identified emotion‐related network patterns (e.g., default mode‐limbic interactions), methodological gaps hinder exploration of spatiotemporal neural oscillations (e.g., millisecond‐scale limbic‐cortical coupling). Key questions remain unresolved, such as real‐time interactions between deep limbic regions (Amy, ACC) and cortical areas during negative emotion induction.

To address these challenges, we employed stereoelectroencephalography (SEEG), which combines millisecond temporal resolution with millimeter spatial precision via intracranial electrodes. This approach enables whole‐brain tracking of neural oscillations and functional connectivity changes during emotion induction. Our study systematically investigates time‐frequency dynamics and network synchronization in cortical‐subcortical structures—a novel contribution to affective neuroscience.

## 2 Materials and Methods

### 2.1 Criteria for Inclusion and Exclusion

A total of 12 patients with drug‐resistant epilepsy who underwent SEEG implantation were included in this study for clinical evaluation. The inclusion criteria were as follows: (1) patients aged between 12 and 65 years; (2) normal cognitive function, defined as a Mini‐Mental State Examination (MMSE) score of ≥ 24 and the ability to accurately comprehend experimental instructions; (3) stable mental health status, indicated by a Hamilton Anxiety Scale (HAMA) score of ≤ 14 and a Hamilton Depression Scale (HAMD) score of ≤ 7. Patients who failed to meet the aforementioned criteria were excluded from the study. Table 1 summarizes the demographic and clinical characteristics of the included patients. All participants provided written informed consent and retained the right to withdraw from the study at any time. This study was approved by the Human Research Ethics Committee of the General Hospital of the Western Theater Command of the People's Liberation Army and conducted in accordance with the principles of the Declaration of Helsinki.

**TABLE 1:** Patient demographics.

ID	Gender	Age (years)	Medical history	Dominant hand	Epileptogenic zone
P1	F	24	22	R	Temporal lobe
P2	F	38	20	R	Temporal lobe
P3	M	24	3	R	Insular lobe
P4	M	41	28	R	Multifocal
P5	M	31	3	R	Parietal lobe
P6	F	35	4	R	Parietal lobe
P7	M	17	3	R	Parietal lobe
P8	M	22	14	R	Multifocal
P9	M	25	8	R	Frontal lobe
P10	M	25	1	R	Temporal lobe
P11	F	14	7	R	Occipital lobe
P12	F	19	18	R	Parietal lobe

### 2.2 Experimental Procedure

**FIGURE 1:**
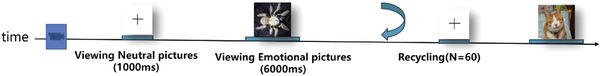
A Standardized Paradigm for Visual Emotional Stimulation. The experiment commenced with the presentation of a neutral cross image, which was displayed for 1 s to establish fixation. Subsequently, various emotional images were randomly presented for a duration of 6 s each. This sequence was repeated for a total of 60 cycles (no identical image appeared more than once within the experiment).

Based on the Chinese Affective Picture System (CAPS), 30 negative and positive pictures were selected to construct an emotion‐evoked atlas using valence and arousal scores. Simultaneous acquisition of SEEG data was achieved through a customized environmental stimulation test and device labeling program, ensuring precise synchronization between image presentation and neural signal recording. The experimental procedure was as follows: participants initially rested with their eyes closed for 1 s, followed by fixation on a neutral cross (used as a baseline) for 1 s. Emotional pictures (500 × 400 pixels, displayed on a 24‐in. screen at a viewing distance of 45 cm) were then presented randomly for 6 s. This sequence was repeated 60 times according to a cyclic mechanism ranging from 1 to 6 s (Figure 1), thereby minimizing interference caused by repeated exposure.

### 2.3 Experimental Data Recording and Pretreatment

Stereotactic surgery was conducted to implant multi‐contact deep intracranial electrodes (Huake‐SDE, 8–16 contacts, 1 mm electrode diameter, 1.5 mm contact spacing). Neurophysiological signals were recorded using a 256‐channel EEG acquisition system (Natus Medical, San Carlos, California, USA) at a sampling frequency of 2048 Hz. All experimental data were preprocessed using a custom program developed on the MATLAB platform. The preprocessing steps included: (1) downsampling the raw EEG signal to 500 Hz; (2) applying a zero‐phase digital filter for bandpass filtering within the range of 0.1–195 Hz; (3) utilizing a notch filter to address power line interference at 50 Hz and its harmonic components; and (4) performing bipolar referencing between adjacent contacts to mitigate volume conduction artifacts. Data were segmented into epochs from stimulus onset to 6 s post‐stimulus and categorized by emotional valence into two groups: Negative Emotion Group (Neg) and Positive Emotion Group (Pos). Epochs containing interictal epileptiform discharges (> 250 ms paroxysmal activity, confirmed by clinical experts) were excluded from further analysis.

### 2.4 Electrode Positioning

**FIGURE 2:**
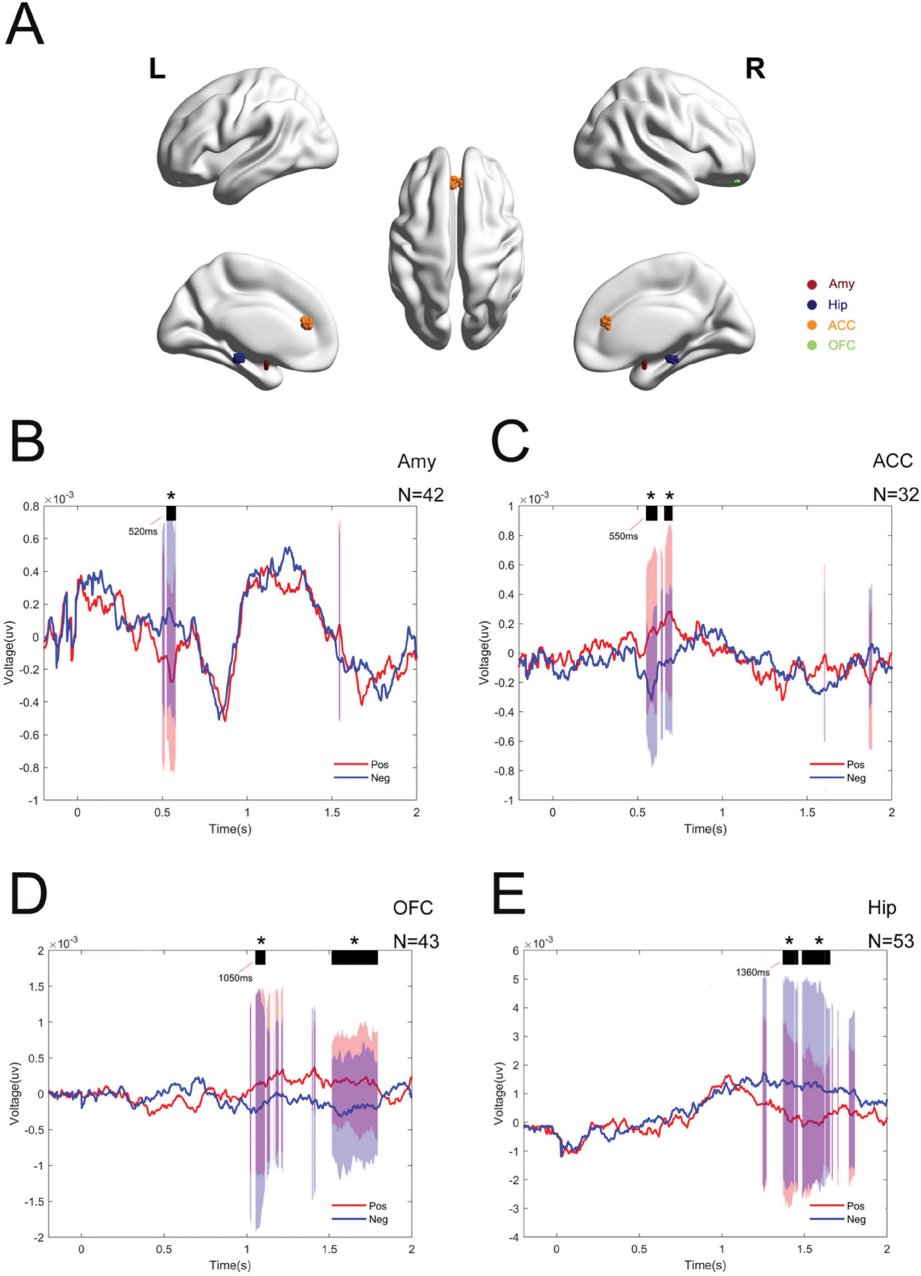
Temporal difference distribution of brain regions related to emotional stimulation. (A) Mapping of electrode contacts located in the Amy, ACC, OFC, and Hip was performed in MNI space. (B) The Amy exhibits a response approximately 520 ms after image stimulation, neural activity characterized by an upregulation of negative emotions. (C) Approximately 550 ms after stimulus onset, the ACC begins to engage in emotional processing, manifested as the downregulation of negative emotions. (D) The OFC is activated around 1050 ms, maintaining a similar pattern of neural activity differences observed in the ACC. (E) Hippocampal activation at 1360 ms reflects upregulation of negative emotions. The average level of the 12 subjects, with statistical significance: *p < 0.05 (> 30 ms duration).

**FIGURE 3:**
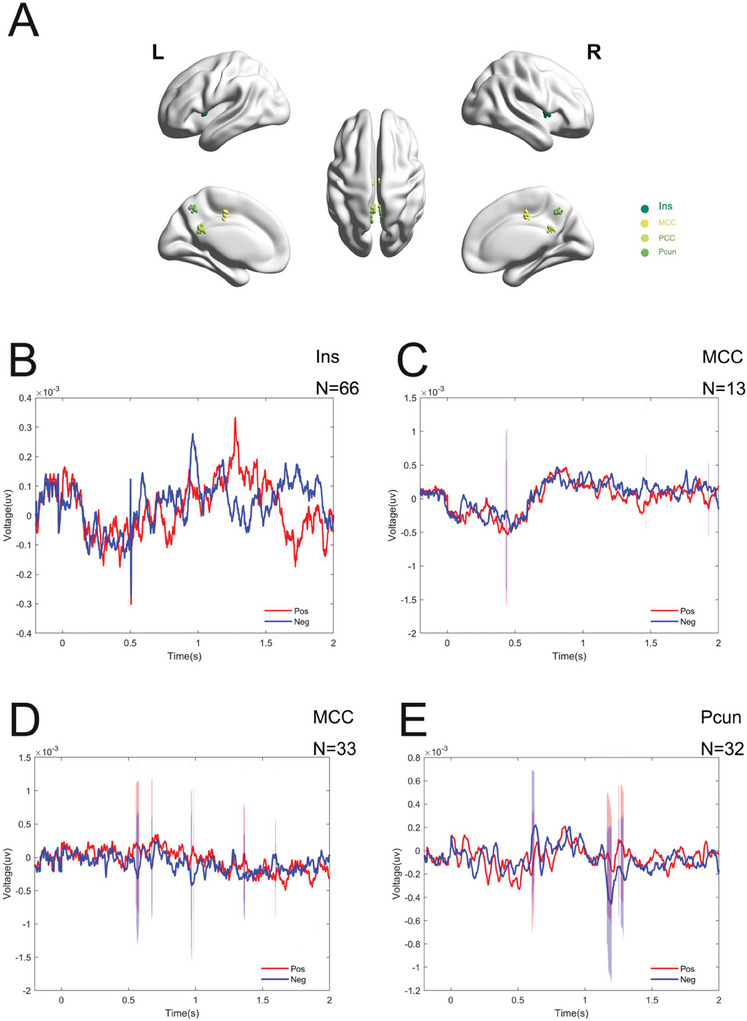
(A) Mapping of electrode contacts located in the Ins, MCC, PCC, and Pcun was performed in MNI space. (B–E) No significant between‐group differences in neural activity were observed in the Ins, Pcun, MCC, or PCC in response to emotional picture stimulation.

In this study, the Brain Storm software was utilized to integrate and co‐register preoperative magnetic resonance imaging data with postoperative computed tomography data. The intracranial electrodes were mapped onto the standardized Montreal Neurological Institute (MNI) brain space coordinate system (Takehara et al. 2020). To ensure localization accuracy, the registered and normalized fused images were independently reviewed by two experienced clinicians. The Regions of Interest (ROI) selected for this study were based on emotion‐related brain regions reported in previous studies, including the Amy, ACC, middle cingulate gyrus (MCC), posterior cingulate gyrus (PCC), Hip, insular lobe (Ins), precuneus (Pcun), and OFC (Kragel et al. 2018; Skelin et al. 2019; Misquitta et al. 2021; Medalla et al. 2022). Subsequently, the BrainNet Viewer software was employed to spatially map and visualize the electrode contacts according to their calculated anatomical coordinates (Csermely et al. 2013). A total of 12 subjects were included in this study. After verification of electrode contact locations, the distribution of electrode contacts across brain areas was as follows: 55 contacts in the Amy, 55 contacts in the ACC, 26 contacts in the MCC, 45 contacts in the PCC, 77 contacts in the Hip, 87 contacts in the Ins, 71 contacts in the OFC, and 42 contacts in the Pcun (Figures 2 and 3).

### 2.5 Experimental Analysis Methods

#### 2.5.1 Event‐Related Potential

This study employed Event‐Related Potential (ERP) analysis to investigate temporal differences in local field potentials (LFPs) during emotional picture processing. LFPs were recorded for 2000 ms post‐stimulus. Signals were baseline‐corrected, normalized, and analyzed using Fast Fourier Transform (FFT) to compute trial‐averaged ERPs. Paired *t*‐tests compared negative and positive emotional conditions, with significant differences defined by both statistical threshold (*p* < 0.05) and temporal consistency (> 30 ms duration). This approach balances statistical rigor with temporal precision, aligning with standard neuroelectrophysiological protocols.

#### 2.5.2 Power Spectral Density

Power Spectral Density (PSD) analysis was used to investigate frequency‐dependent neural oscillations by quantifying changes in LFP energy distribution across five characteristic bands: delta (0.5–4 Hz), theta (4–8 Hz), alpha (8–13 Hz), beta (13–30 Hz), and gamma (30–100 Hz) (Sonkusare et al. 2020). Emotional images were presented as stimuli to evoke LFPs, with Welch's method applied to estimate the power spectrum of raw signals. Average PSD values were computed for each frequency band and categorized by experimental conditions and groups. To reduce inter‐subject variability, individual lead spectra were normalized by dividing each frequency band's total power by the corresponding lead's power spectrum, generating trial‐averaged normalized PSD values (Chernykh et al. 2022). Paired *t*‐tests (*p* < 0.05 significance threshold) were conducted to statistically compare spectral characteristics between negative and positive emotional stimuli within each frequency band, revealing dynamic modulations in brain network activation patterns under different emotional states.

#### 2.5.3 Phase Locking Value

Cognitive processes can be characterized not only by the time‐frequency activity patterns of various brain regions but also by the information transmission and dynamic interactions between different functional areas. In contrast to time‐frequency analysis methods, connectivity analysis places greater emphasis on quantifying the functional coupling among multiple brain regions, thereby partitioning the brain into several functionally interconnected neural circuits. In synchronous activity analysis, we construct a synchronization measurement matrix to capture the synchronous neural activity in the brain, enabling a deeper exploration of structural networks, functional connectivity, and effective cognitive modes involved in diverse emotional experiences. In this study, we first applied the Hilbert transform to filtered LFPs to extract instantaneous phase information from the signals. Subsequently, we calculated the instantaneous phase differences between paired signals and quantified the Phase Locking Value (PLV) using the absolute value of the phase difference index (Mercier et al. 2022). To more precisely compare PLV differences across experimental conditions, we further adjusted for baseline period phase‐locking values. Phase‐locking values range from 0 (completely random) to 1 (perfectly phase‐locked).

## 3 Results

### 3.1 Investigating Temporal Variations in Task‐Related SEEG Signals

In neural information processing of emotional picture stimulation, each brain region exhibits distinct temporal dynamics and functional specificity. Specifically, ERP analysis revealed that Amy activation initially occurs approximately 520 ms after stimulus onset, marked by an increase in neural activity associated with negative emotional processing. Subsequently, around 550 ms after stimulus onset, the prefrontal cortex became involved in emotional processing, as indicated by a reduction in neural activity linked to negative emotional responses. As neural information propagates further, OFC is activated around 1050 ms, maintaining a similar pattern of neural activity differences observed in the ACC. Notably, at 1360 ms, the Hip re‐exhibits neural activity characteristics akin to the initial activation of the Amy (Figure 2). However, within the 0–2 s time window, other brain regions such as the Ins, Pcun, MCC, and PCC do not demonstrate significant neural activity differences in response to emotional pictures (Figure 3). These findings suggest that emotional information processing may rely on a hierarchical activation pattern across specific brain regions, and the temporal dynamics of different brain regions during emotional processing may reflect their specialized roles in the encoding, evaluation, and integration of emotional information.

### 3.2 Frequency‐Domain Characteristics of Electrophysiological Signals in Emotion‐Related Brain Regions

**FIGURE 4:**
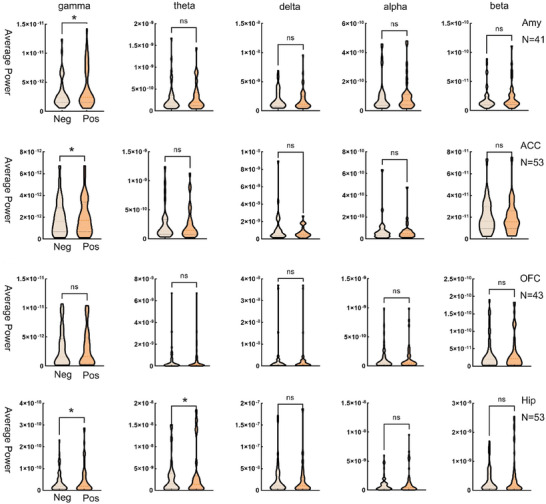
Power changes of emotion‐related brain regions. Arranged from top to bottom, the Amy, ACC, and Hip exhibited significantly lower gamma power in the negative emotion group (*p < 0.05). In contrast, OFC demonstrated no significant differences across the entire frequency band.

After emotional picture stimulation, the Amy, ACC, OFC, and Hip were identified as new ROI through the analysis of response patterns across different brain regions within a specified time window. PSD analysis revealed that in the gamma band, Amy (gamma: *p* = 0.002; delta: *p* = 0.367; theta: *p* = 0.354; alpha: *p* = 0.821; beta: *p* = 0.072), Hip (gamma: *p* = 0.027; delta: *p* = 0.358; theta: *p* = 0.031; alpha: *p* = 0.111; beta: *p* = 0.258), and ACC (gamma: *p* = 0.013; delta: *p* = 0.079; theta: *p* = 0.727; alpha: *p* = 0.130; beta: *p* = 0.169) exhibited significant between‐group differences (Neg < Pos, *p* < 0.05). Additionally, Hip demonstrated differences in the theta band. No significant differences were observed in other frequency bands. Notably, although OFC showed potential differences in time‐domain analysis, its PSD did not exhibit significant between‐group differences across all frequency bands (gamma: *p* = 0.553; delta: *p* = 0.600; theta: *p* = 0.241; alpha: *p* = 0.799; beta: *p* = 0.346) (Figure 4). This suggests that the functional response of OFC may be independent of the frequency domain activity patterns observed in other brain regions. These findings underscore the necessity for further investigation into the underlying mechanisms of OFC.

### 3.3 Frequency‐Specific Neural Synchronization in Emotional Network Responses

**FIGURE 5:**
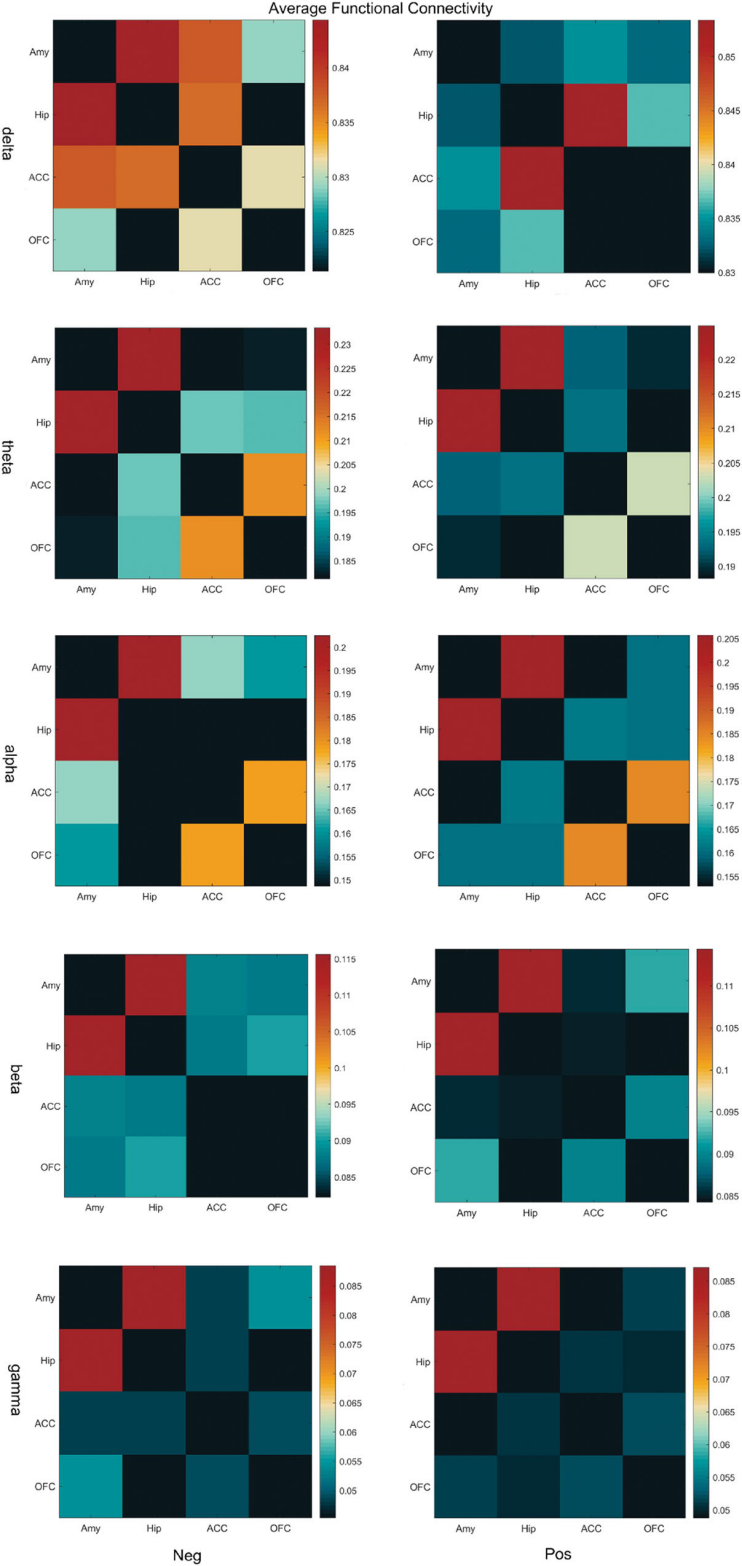
Changes in synchronization between brain regions. During visual stimulation, significant interregional synchronization changes were observed across distinct frequency bands. Notably, exposure to negative emotional stimuli markedly enhanced the mean PLV between the Amy and other brain regions, with particularly pronounced synchronization increases in the delta frequency band.

Human emotional processing involves the dynamic interaction of multi‐level brain functional networks, and its neural mechanism primarily depends on the synchronous oscillatory activity of distributed neural networks. As the spatial integration signal of postsynaptic potentials in neuronal ensembles, phase synchronization of LFPs serves as a critical indicator for investigating the functional integration of brain neural electrical activities. Experimental studies have demonstrated that, in visual stimulation tasks, enhanced phase synchrony is significantly and positively correlated with stimulation‐induced large‐scale brain functional network integration (Q. Li et al. 2017). Our results indicate that negative emotional picture stimulation significantly increases the average phase locking between the Amy and other brain regions across different oscillation frequency bands, particularly in the delta frequency band. A similar trend of enhancement was observed in other frequency bands (Figure 5). These findings suggest that neural oscillations in the delta band may play a specific functional role in the neural circuitry underlying negative emotions.

## 4 Discussion

This study utilized SEEG to systematically record neural activity in deep brain regions during emotional processing. Negative emotional stimuli significantly activated limbic structures, including the Amy, ACC, Hip, and OFC, inducing multi‐band oscillatory dynamics. ERP and PSD analyses revealed distinct spatiotemporal activation patterns, while PLV metrics highlighted enhanced functional connectivity within these regions under negative emotional conditions. These findings support the hypothesis that negative emotion processing relies on dynamic interactions within distributed neural networks.

### 4.1 Amy's Early Role in Negative Emotion Processing

Amy exhibited the earliest response to negative stimuli (550 ms post‐stimulation), aligning with EEG studies showing sustained engagement via late positive potentials (LPP) (Yang et al. 2019). This rapid activation underscores its role in detecting salient threats and initiating downstream physiological responses (e.g., autonomic arousal) through hypothalamic and brainstem projections (Kim et al. 2011). Preclinical studies further link Amy hyperactivity to anxiety‐like behaviors via synaptic plasticity in the basolateral Amy (Ganzel et al. 2007). Anatomical connections between the Amy and primary sensory cortices suggest it prioritizes unprocessed threat signals, enabling immediate survival‐oriented reactions (Rolls 2015).

### 4.2 OFC in Emotion Regulation

The OFC displayed delayed activation (∼1050 ms), reflecting its role in value‐based appraisal and top‐down modulation of Amy activity. As a multimodal integration hub (Stein et al. 2007), the OFC assigns cognitive labels to stimuli, suppressing excessive negative responses when threats are deemed non‐critical. This interaction facilitates adaptive decision‐making by reconciling historical and current reward valuations, thereby minimizing behavioral errors (Pujara et al. 2022). The temporal lag between Amy and OFC activation highlights a bottom‐up‐to‐top‐down processing hierarchy in emotion regulation.

### 4.3 Cingulate Cortex Integration of Emotion and Cognition

The ACC showed intermediate activation (∼550 ms), integrating bottom‐up Amy inputs with top‐down OFC evaluations to guide action‐outcome learning. Enhanced ACC activity correlated with negative emotion downregulation, suggesting its role in balancing stimulus salience and contextual appraisal. However, the absence of defensive behaviors in this paradigm implies that non‐threatening stimuli may fail to trigger ACC‐driven action plans, consistent with its function in cost‐benefit analysis (Kolling et al. 2016).

### 4.4 Hippocampal Modulation of Emotional Memory

Hip activation (∼1360 ms) coincided with intensified negative responses, likely reflecting noradrenergic‐mediated enhancement of episodic memory encoding. The Amy‐Hip circuit may prioritize retention of emotionally salient events by modulating attention and perceptual encoding (Amaral et al. 2003; Hagena et al. 2016; Bacon et al. 2020). Pharmacological evidence supports noradrenaline's role in strengthening emotional memory consolidation, though direct human measurements remain challenging.

### 4.5 Frequency‐Domain Dynamics and Network Synchronization

Negative emotions suppressed gamma‐band power in limbic regions, potentially due to disrupted GABA‐glutamate balance and resource reallocation toward default mode network activity (Gonzalez‐Burgos et al. 2011; Jombik et al. 2016; Hossain et al. 2018). Conversely, delta‐band PLV increased in the Amy‐ACC‐Hip‐OFC network, indicating enhanced low‐frequency synchronization for cross‐regional integration of emotional information (Lindquist et al. 2016; Dang et al. 2022). These frequency‐specific changes underscore the trade‐off between localized processing efficiency and global network coordination during emotional states.

## 5 Conclusion

By combining SEEG with standardized emotional paradigms, this study delineated the spatiotemporal dynamics and network interactions underlying negative emotion processing. Amy's early threat detection, OFC's regulatory appraisal, ACC's integrative evaluation, and Hip's memory modulation collectively illustrate a distributed neural mechanism. These findings bridge electrophysiological and neuroimaging evidence, advancing models of affective circuitry. In the brain ROI, such as the insula, the expected neural responses were not observed. This suggests that although electrode placement was verified histologically, minor positional inaccuracies may have prevented access to the target functional subregions. Alternatively, the absence of activation could be due to the task paradigm's inability to sufficiently engage the relevant neural circuits or the limited involvement of this region in the behavior under investigation. Future work should employ dynamic network modeling and multimodal fusion to unravel the real‐time interactions governing emotional processing.

## Author Contributions

**Zheng Li**: writing – original draft, project administration, data curation. **Rong Yang**: visualization, validation. **Jiaxi Zhao**: data curation, project administration. **Kanglin Liu**: data curation, project administration. **Sixun Yu**: writing – review and editing, conceptualization, supervision. **Xin Chen**: Software, writing – review and editing, methodology. **Haifeng Shu**: Conceptualization, writing – review and editing, funding acquisition, supervision.

## Ethics Statement

This study received approval from the Human Research Ethics Committee of the General Hospital of the Western Theater Command of the People's Liberation Army.

## Consent

All participants provided written informed consent in line with the standard ethical guidelines of the Declaration of Helsinki.

## Conflicts of Interest

The authors declare no conflicts of interest.

## Peer Review

The peer review history for this article is available at https://publons.com/publon/10.1002/brb3.70951

## Data Availability

The data that support the findings of this study are available from the corresponding author upon reasonable request.
